# Measuring design thinking competence in Taiwanese nursing students: a cross-cultural instrument adaptation

**DOI:** 10.1186/s12909-023-04911-z

**Published:** 2023-12-08

**Authors:** Hsing-Yuan Liu

**Affiliations:** 1 Department of Nursing, Chang Gung University of Science and Technology, Taoyuan City, Taiwan, ROC; 2 Nursing Department, New Taipei Municipal TuCheng Hospital (Built and Operated by Chang Gung Medical Foundation), New Taipei City, Taiwan; 3 Department of Nursing, Chang Gung University, Taoyuan City, Taiwan

**Keywords:** Creative synthesis inventory design thinking competence, Nursing students, Taiwanese

## Abstract

**Background:**

Design thinking (DT) competence, a creative problem-solving ability, has been investigated extensively among college students in various professional fields within the western cultures. No instrument, however, is available for assessing DT competence in nursing students, particularly, those in non-western cultures.

**Objective:**

To adapt and validate the use of Creative Synthesis Inventory (CSI) assessing the four components (i.e., visualization, discovery, prototyping, and evaluation) of DT competence in nursing students in Taiwan.

**Design:**

Cross-sectional, mixed methodological design combining qualitative and quantitative strategies.

**Participants:**

A 19-item CSI was administered to a total of 520 nursing students from two campuses of a science and technology university in Taiwan. The data collection was conducted between September 2020 and June 2022.

**Methods:**

The cross-cultural adaptation and validation of CSI-Taiwan was done in two phases: Phase I included content validity check, translation, and cross-cultural adaptation. Phase II involved pilot testing and psychometric evaluation.

**Results:**

A first-order confirmatory factor analysis validated the four-component structure, namely visualization, discovery, prototyping, and evaluation, of an 18-item CSI-Taiwan (model fit indices: χ^2^/df = 3.953, NNFI = 0.942, CFI = 0.956, TLI = 0.947, SRMR = 0.035, and RMSEA = 0.075). A second-order confirmatory factor analysis further indicated that the four components converged on a unitary construct of DT competence (model fit indices: χ^2^/df = 4.082, NNFI = 0.935, CFI = 0.949, TLI = 0.940, SRMR = 0.040, and RMSEA = 0.080). Moreover, the CSI-Taiwan also demonstrated satisfactory reliability and validity. Together these results validated the construct of DT competence and its components as theorized.

**Conclusions:**

The CSI-Taiwan was a reliable and valid self-report instrument to be used by Taiwanese nursing students.

**Supplementary Information:**

The online version contains supplementary material available at 10.1186/s12909-023-04911-z.

## Introduction

The concept of design thinking (DT), often applied a human-centered approach to solve problems that has subverted the traditional models of business innovation. Creative and critical thinking coupled with interdisciplinary team collaboration brings not only innovations in services, but also improvements of products [1]. DT has also been employed to solve the multiple complicated issues in medical management and innovation [2, 3].

Although DT has been viewed as a successful tool for teaching creativity in higher education, the application of DT to nursing education is not broadly explored [4]. As a result, DT competence in nursing students has rarely been the target of research investigation [5]. Validating instruments for measuring the DT competence in nursing students are also lacking [6]. More importantly, previous research on DT competence in college students has been primarily done in the western cultures [7]. Related empirical investigations in the non-western cultures are scant [8]. To fill this knowledge gap, this study investigated the cultural adaptation and validation of the Creative Synthesis Inventory-Taiwan, a questionnaire evaluating DT competence that is in agreement with the local culture and nursing educational context.

## Background

### Design thinking

DT is a human-centered innovation process highlighting visualization of ideas, fast learning, collaboration, and rapid concept prototyping, which has a strong influence on business innovation and stratification [9]. According to Brown [10], DT has also been widely viewed as a tool that helps organizations create a competitive advantage by formulating solutions that meet consumer needs. Although there is no agreement on a single definition [11], in a recent review of DT in education, Lor [12] concluded that DT involves a dynamic non-linear process. Broadly defined, DT is seen as an iterative approach to problem solving that requires empathizing with needs of others, thinking interactively, integrating advice from others, producing various ideas, testing out potential solutions by creating prototypes, and learning from failure to reach a solution [1].

### Design thinking in healthcare education

Nursing staff stands in the front-line on patient care as well as in the dominating position of evaluating and implementing process of DT ideation. They need to collaborate with interdisciplinary healthcare professionals and redesign the healthcare system in order to face the constantly changing environment in the current medical system [13, 14].

Research has also used DT as a learning and teaching method in higher education [15, 16]. In education, DT refers to “an orientation to learning that comprises active problem solving and marshaling one’s capability to generate impactful change” [12]. As DT has become increasingly important in higher education, promoting college students’ DT competence, therefore, has gained a lot of attention. In the field of healthcare, most of the empirical studies conducted in the United States investigated the effectiveness of applying the concept of DT [7]. Only a few studies have focused on nursing students [6], especially those in non-western cultures [8].

Since 2003, the Advisory Office of the Ministry of Education in Taiwan has declared fostering creativity in students at all education levels as a priority [17]. According to Liu et al. [17], five Taiwanese nursing schools have integrated DT into capstone courses. Nonetheless, research on nursing students’ DT competence is largely absent from the literature. To the best of our knowledge, only one study investigated nursing students’ DT competence in Taiwan [14].

### Measuring design thinking competence

There is a lack of agreement on how to measure DT competence in the process for innovation. Badding [9] constructed a quantitative instrument measuring attributes and factors contributing to increased innovation within DT environments. Through quantitative testing, she confirmed a four-component structure in the DT process, namely, visualization, discovery, prototyping, and evaluation. Visualization refers to the capability to imagine an end result of a product or the process that is essential to how designers work and think [18]. Discovery is defined as developing a profound understanding of a product or user through involvement, observation, innovative thinking, and user assessment [9]. Prototyping is viewed as the skill of generating ideas turning into actual services and products to be tested, iterated, and refined [9]. Evaluation involves an iterative process to examine prototypes and elaborate problem solutions before the idea is viewed as finished [9]. In order to understand Taiwanese nursing students’ abilities in innovative problem solving, this study focused on these four components in the DT process [9, 14].

While quantitative instruments have been developed or adapted as measures of DT competence [9, 19], all were in English and none was developed for the professional field of healthcare. Given the lack of quantitative measurements for nursing students, this study aimed at developing a reliable and valid questionnaire for assessing DT competence in Taiwanese nursing students. Therefore, this study was to adopt and modify an existing questionnaire, Creative Synthesis Inventory (CSI), determine its psychometric properties, and validate its use with Taiwanese nursing students.

## **Methods**

### Design

**Fig. 1:**
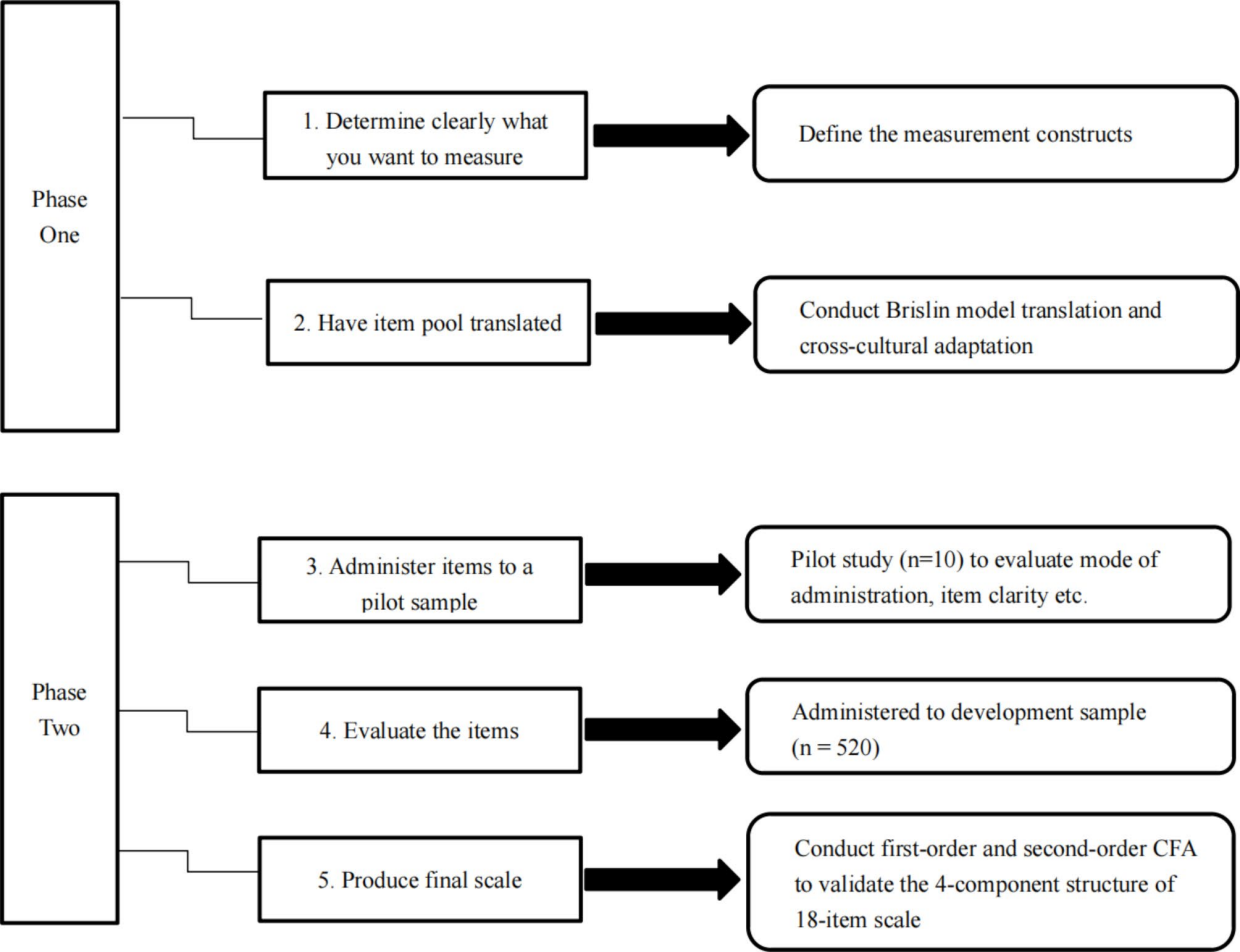
Two-phase research procedure based on Weathers et al. (2020)

To measure DT Competence in Taiwanese nursing students, this study used a cross-sectional, mixed methodological design combining qualitative and quantitative strategies, which was conducted in two phases with five steps (Fig. 1). Phase I focused on content validity check and item examination of the measurement. After the measurement constructs were defined (step 1), item translation and cross-cultural adaptation for the scale was implemented (step 2). Phase II focused on empirical examination of the translated measurement, including pilot testing of the intial measurement (step 3) and psychometric evaluations of the initial and revised measurement (steps 4–5),. Following construct validity check of the initial measurement, convergent and discriminant validity check as well as reliability check of the revised measurement were performed. See below for the detailed description of each research step.

#### Phase I: item examination and translation

*Step 1: Define the measurement constructs*

In order to build a measurement tool, the research team first started by identifying the constructs that compose the DT competence based on Badding’s model [9]. According to Badding, DT competence has been discussed as creative synthesis process whereby elements and characteristics contributing to innovation, including: visualization, discovery, prototyping, and evaluation [9]. As reviewed in the Introduction, visualization is regarded as the integrating mental images that can be manipulated, and the skill with which a person is able to recognize essential attributes of new combinations of things [9, 20]. According to Curedale [20], discovery is defined as developing a profound understanding of a product or user through involvement, observation, innovative thinking, and feedback; listening and understanding; and then explaining viewpoints of problems and needs. Brown and Wyatt [21] defined prototyping as a method to assess possible consequences and potential future success of a design idea [9]. Prototyping creates a context where ideas take physical shape, allowing the best ideas visualized and imagined to be tested, iterated, and elaborated before moving into the assessment stage [9, 22]. Martin and Christensen [23] viewed evaluation as a process providing the person or design team with an iterative process to test prototypes and elaborate the problem solutions before the idea is viewed as finished [9].

In Badding’s 19-item CSI, 5 items are used to measure visualization, 4 items measure discovery, 4 items measure prototyping, and 6 items measure evaluation. In this study, a five-point Likert scale scoring from 1 (strongly disagree) to 5 (strongly agree) was used. Higher scores indicated higher perceived levels of DT competence. Cohen et al. [24] suggested that the scale was structured in a manner that increased readability and no difficulty in completion [25]. Three instructors from the industrial design department provided their feedback on the face validity of the questionnaire items.

*Step 2: Brislin translation and cross-cultural adaptation*

**Fig. 2:**
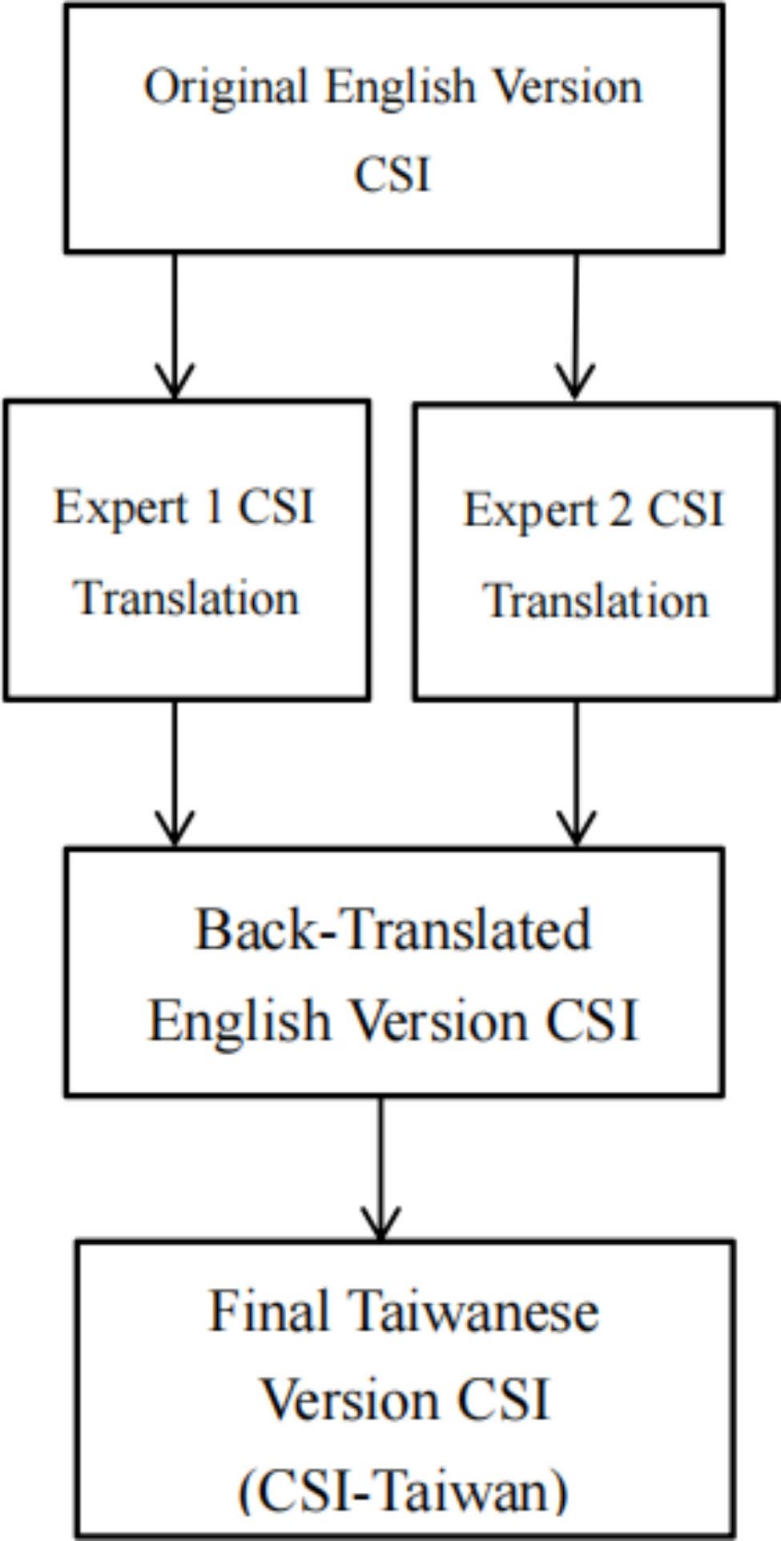
Brislin’s translation flow process

On the basis of Brislin [26] and Guillemin et al., [27], the forward- and backward-translation between the Taiwanese and English versions of the CSI was done with a cross-cultural adaptation process to ensure the cultural appropriateness and equivalence. The Brislin translation process was carried out as follows: (1) Two translators blind to the literature independently conducted forward translation; (2) Synthesis of the two translations was used by the research group; (3) A bilingual professional translator who was blind to the original English version back-translated the Taiwanese version to English; and (4) The research group compared the back-translated version with the original version. The lack of discrepancy between the versions indicated satisfactory translation. Thus, the Taiwanese version of the CSI (CSI-Taiwan) was obtained. The process of the Brislin translation is displayed in Fig. 2.

Additionally, a cross-cultural adaptation process was conducted that included expert committee review, focus group discussion, and a pilot test recommending by Guillemin et al. [27]. In the expert committee review, the Taiwanese version of CSI was reviewed by an expert panel. In addition to 3 nursing experts, the panel consisted of one expert with expertise in DT from the industrial design department who identified translation discrepancies with the CSI original version and thoroughly discussed cultural barriers. After consensus was reached by these experts, the second version of the Taiwanese CSI was developed. All the items in CSI-Taiwan had a Content Validity Index (CVI) of 0.8 or greater meeting the requirement [28]. In the focus group discussion led by the author, the second version of the translated questionnaire was discussed among 3 nursing faculty members and 1 industrial design faculty member, and 1 nursing supervisor of a teaching hospital. Data from the two hours of the focus group discussion was collected, transcribed and analyzed. Findings were combined with the recommendations from the expert panel review. A pilot test with a bilingual version of the instrument has been recommended to determine the equivalence between the newly translated and the original instruments.

A finalized 19-item survey was administered to 10 students as a pilot study to assess the applicability and cultural acceptability of CSI-Taiwan.

#### Phase Џ: pilot testing and psychometric evaluation

The second Phase was conducted in three steps, including pilot testing, items evaluation, and final scale producing.

*Step 3: Pilot testing*

This pilot testing was conducted with nursing and industrial design students (n = 10) from two science and technology universities in Taiwan. Students reported no problems with the instrument. In the pilot test, the Cronbach’s alpha was 0.78 indicating that internal consistency of the scale was good. The results of the pilot testing were considered satisfactory and the final version of CSI-Taiwan was obtained.

*Steps 4 and 5: Items evaluation and final scale producing*

The final version of 19-item CSI-Taiwan was administered to a larger sample of nursing students for psychometric evaluations. First, construct validity was assessed through confirmatory factor analysis. Reliability, convergent validity, and discriminant validity of the CSI-Taiwan were also evaluated.

### Participants and procedure

Nursing students (n = 520) working on completing the second year of their 2- (*n* = 180) or 4-year (*n* = 340) program at the college of nursing participated in this study. The students were recruited from the capstone course that was part of the nursing program. Capstone courses serve as the culminating learning experience for students nearing the end of their college programs. To provide a closure to students’ learning and prepare for their looming transition from school to work, the main purpose of capstone courses is to help students integrate, consolidate, and reflect on their learning experience [29].

The capstone course in this study was designed for students nearing the end of their programs. The goal of the capstone course was to foster students’ ability to discover healthcare problem through healthcare practices, and apply learned skills through capstone projects. The practical experience in the analyses and understandings of problems as well as the development of healthcare-related innovative products were expected to enhance students’ DT competence and innovative abilities.

The 18-week long course was offered to both industrial design and nursing students at the two campuses of a university of science and technology in Taiwan. The course was co-taught by the instructors from industrial design and nursing departments. The course emphasis was on creativity and collaboration requiring students to work collaboratively in teams and design a prototype of a healthcare product that has the potential to be patented. At the end of the course, each student team made a presentation on their design. In the capstone course, nursing students were expected to learn fundamental theories and connotations of DT and collaboration skills, deploy “Visualization” skills to discover and define problems of product users’ issues, and design healthcare product prototypes through brainstorming. Finally, they invited the stakeholders to test and evaluate the prototypes [14, 30]. The data collection with nursing students was conducted from September 2020 to June 2022.

### Measures

After obtaining the permission from the original author for translating the CSI scale into Chinese for a cultural adaptation study, a 19-item self-report questionnaire, CSI-Taiwan, was administered to assess nursing students’ perceptions of their DT competence in four dimensions, including: visualization, discovery, prototyping, and evaluation. The CSI-Taiwan questionnaire began with the phrase, “In the nursing capstone implementation process, I can…”. Each item was scored on a five-point Likert scale, ranging from 1 = strongly disagree to 5 = strongly agree. All 19 items of CSI-Taiwan are shown in Table 1. Information on the demographic characteristics of the participants, including age and gender, was also collected.

**Table 1:** The 19 items of the Creative Synthesis Inventory-Taiwan.

Items In the nursing capstone implementation process, I can:
Q1: Convert reality into an abstract thought or idea
Q2: See connections among your ideas
Q3: Mentally manipulate ideas into new combinations
Q4. Draw ideas without using words or numbers
Q5.Think creatively
Q6: Acknowledge other’s diverse knowledge for potential application in solution(s)
Q7. Allow yourself to be open to discovery of new ideas
Q8. Utilize team decision-making
Q9. Test and define to gain feedback and learn rapidly
Q10. Create mock-up(s) (e.g., a physical to scale replica or prototype created and tested before problem/project is finalized)
Q11: Explore feasibility of your mock-up(s) (e.g., a physical to scale replica or prototype)
Q12. Discover end-use possibilities from preliminary mock-up(s)
Q13. Judge the impact of the mock-up(s) to achieve design concept
Q14. Assess project qualities (i.e., market potential, alignment to strategic goals, competitive advantage)
Q15. Receive external feedback from stakeholders/client(s)
Q16. Measure quality from end-user perspective, seeking to understand usefulness (e.g., value intention, perception, ease of use, and universal application)
Q17. Judge the level of innovation
Q18. Use a variety of data collection approaches to seek information from client and/or end-user
Q19. Define measures of success (e.g., project objectives, problem solutions, and return on investment)

### Statistical analysis

First- and second-order confirmatory factor analysis (CFA) was performed to assess CSI-Taiwan’s construct validity. CFAs were conducted to confirm the 4-factor structure as theorized by Badding [9]. Goodness-of-fit indices were employed to determine the model fit. First, the chi-square (χ^2^) test was used to assess the absolute fit of the model. Since it can be resulted in false positives [31], the chi-squared value/degree of freedom (χ^2^/df) was used instead. A value of less than 5.0 is considered satisfactory. Five more additional indices of goodness of fit were also used, including: non-normed fit index (NNFI), comparative fit index (CFI) and Tucker-Lewis index (TLI), standardized root means squared residual (SRMR), and root mean square error of approximation (RMSEA).

To establish the convergent validity of CSI-Taiwan, composite reliability (CR) indexed by omega (ω) and the average variance extracted (AVE) need to be considered. CR indicates the extent to which individual items are related to a specific factor. Briefly, CR is the variance due to the factor divided by the total variance of the composite, i.e., the total variance of the sum divided by the variation due to the factor, and the acceptable value of CR is above 0.7. AVE is calculated as the mean of the squared standard loadings of each indicator associated with a latent construct, and it must be 0.50 or greater. Furthermore, to establish discriminant validity square root of AVE and inter-construct correlation coefficients must be considered [32, 33]. On the basis of the Fornell-Lacker criterion, the square root of each dimension’s AVE should have a greater value than its correlation with other latent dimensions [33].

### Ethical considerations

We obtained approval from the Institutional Review Board (IRB) of the hospital ethics committee. The nursing students were invited participate in the study by the author of this study during the last week of the capstone course. Informed consent was provided to all targeted nursing students before their research participation. The students were fully informed that their participation was voluntary and anonymous, that they could withdraw from the research at any time, and that their grades were not affected by their participation or withdrawal.

## Results

### Demographic characteristics

Demographic characteristics of the participants are shown in Table 2. Among the 520 participants, the vast majority were women. The participants whose ages ranging from 20 to 33 years had a mean age in their early twenty. More than half of the students previously participated in the programs related to DT competency improvement. Table 3 shows the descriptive statistics for the individual items of the 19-item CSI-Taiwan based on Badding’s four components.

**Table 2:** Demographic characteristics of the participants (*n* = 520)

Variable	n (%)	Mean (SD)
Gender		
Male	73 (14)	
Female	447 (86)	
Age (years)		22 (1.17)
Previous participation in DT related programs		
Yes	317 (61)	
No	203 (39)	

**Table 3:** Descriptive statistics for the 19-item Taiwanese version of Creative Synthesis Inventory (*n* = 520)

Dimensions and items	Range	Mean (SD)
**Visualization**	0–25	3.79 (0.70)
CSI_1	0–5	3.68 (0.91)
CSI_2	0–5	3.97 (0.74)
CSI_3	0–5	3.88 (0.78)
CSI_4	0–5	3.59 (0.98)
CSI_5	0–5	3.86 (0.82)
**Discover**y	0–20	4.10 (0.74)
CSI_6	0–5	4.13 (0.74)
CSI_7	0–5	4.14 (0.77)
CSI_8	0–5	4.10 (0.79)
CSI_9	0–5	4.03 (1.53)
**Prototyping**	0–20	3.70 (0.80)
CSI_10	0–5	3.57 (0.96)
CSI_11	0–5	3.69 (0.92)
CSI_12	0–5	3.76 (0.84)
CSI_13	0–5	3.79 (0.87)
**Evaluation**	0–30	3.98 (0.68)
CSI_14	0–5	3.87 (0.83)
CSI_15	0–5	3.82 (0.84)
CSI_16	0–5	3.89 (0.85)
CSI_17	0–5	4.69 (0.76)
CSI_18	0–5	3.82 (0.85)
CSI_19	0–5	3.84 (0.83)

### Confirmatory factor analysis

#### First-order confirmation factor analysis

**Fig. 3:**
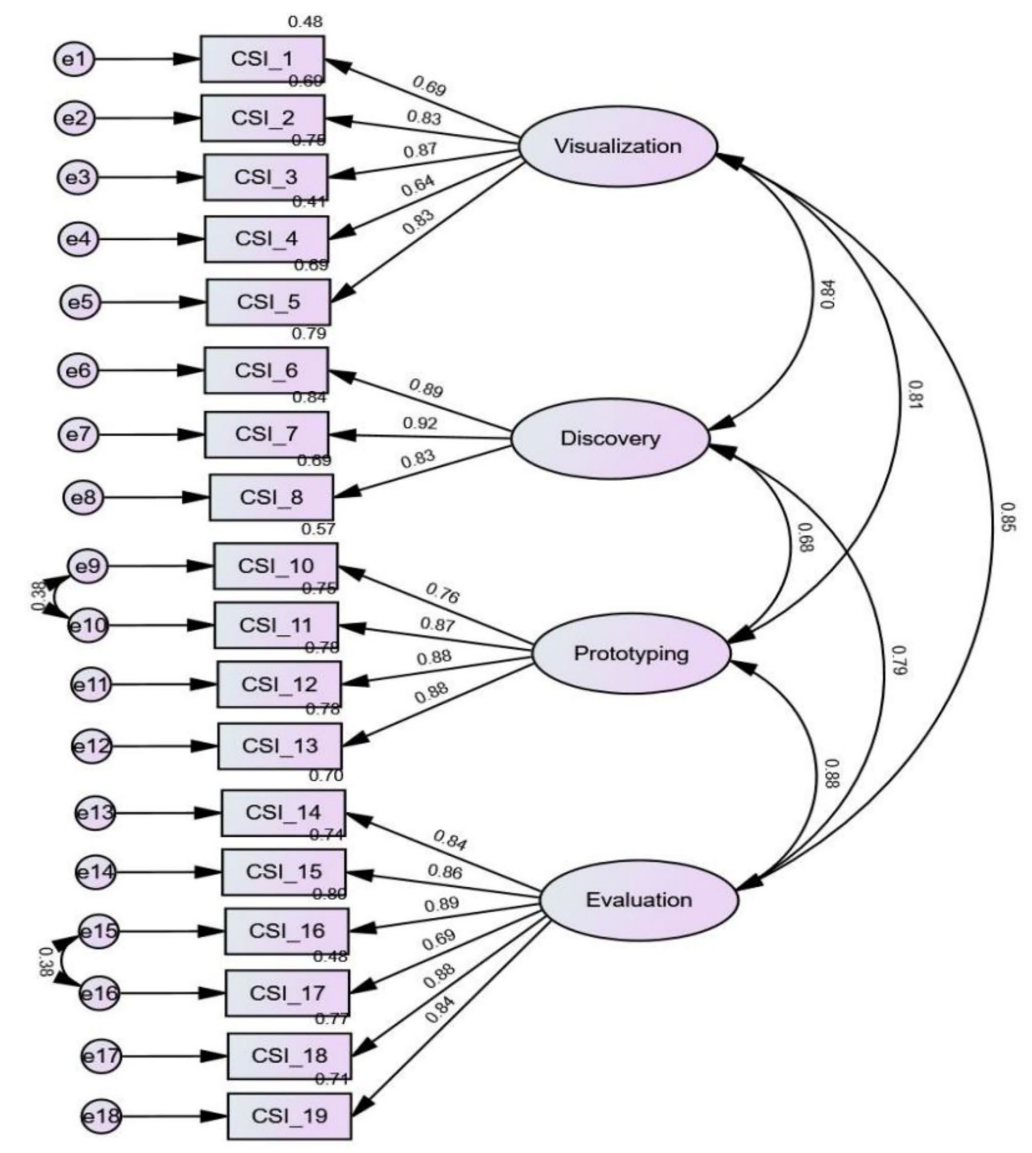
Result of first-order confirmatory factor analysis on the 18-item Taiwanese version of Creative Synthesis Inventory. Note. CSI_1 to CSI_5 denote the questionnaire items loaded on the factor of Visualization; CSI_6 to CSI_8 on the factor of Discovery; CSI_10 to CSI_13 on the factor of Prototyping and CSI_14 to CSI_19 on the factor of Evaluation. Residual errors (e) indicated as follows: e1 to e5 corresponding to the items on Visualization; e6 to e8 to the items on Discovery; e9 to e12 to the items on Prototyping; and e13 to e18 to the items on Evaluation

A first-order CFA was conducted with four correlated latent variables, namely, ‘evaluation’, ‘discovery’, ‘prototyping’, and ‘visualization.’ Examination of the result showed that one item (CSI_9) needed to be removed because its standardized factor loading of 0.33 was lower than the cut-off value of 0.40. In addition, according to the modification indices, two residual covariances were added to improve the model fit. The graphic representation of the revised first-order model with standardized factor loadings for the 18 items is depicted in Fig. 3, which ranged from 0.64 to 0.92, moderate to strong. The goodness-of-fit indices for the model were: χ^2^/df = 3.953, NNFI = 0.942, CFI = 0.956, TLI = 0.947, SRMR = 0.035, and RMSEA = 0.075. Based on the evaluation criteria of RMSEA and SRMR ≤ 0.08 and NNFI and CFI ≥ 0.90 [34, 35]. Together, the model was deemed to have a good fit with the data [36]. Results of the first-order confirmatory factor analysis on the 18-item Taiwanese version of Creative Synthesis Inventory was presented in Fig. 3, which demonstrated a correlated 4-factor model with standardized factor loadings on Visualization, Discovery, Prototyping, and Evaluation, respectively.

#### Second-order confirmation factor analysis

**Fig. 4:**
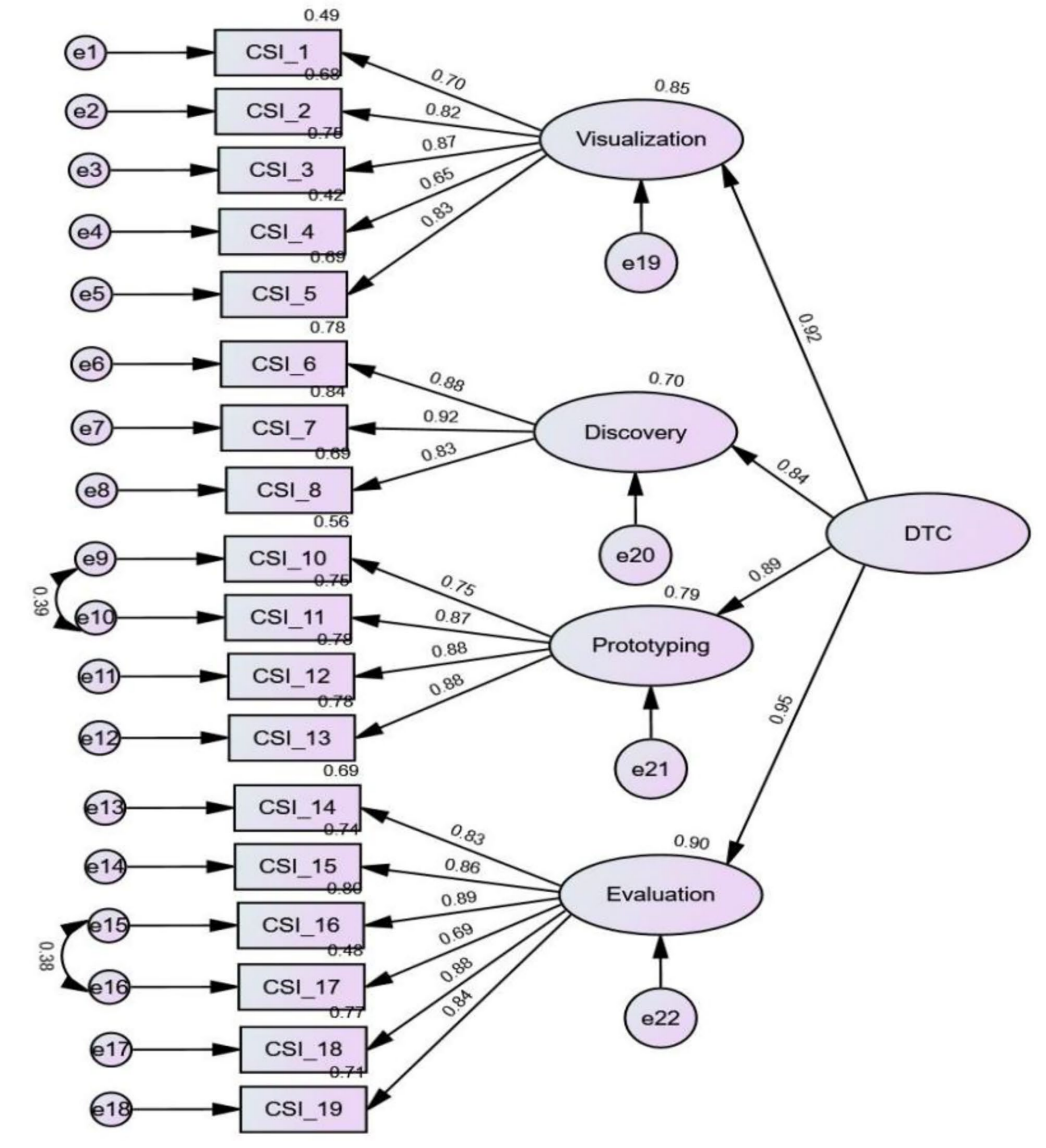
Results of second-order confirmatory factor analysis on the 18-item Taiwanese version of Creative Synthesis Inventory. Note. For simplicity, the correlations between the four latent variables are omitted. CSI_1 to CSI_5 denote the questionnaire items loaded on the factor of Visualization; CSI_6 to CSI_8 on the factor of Discovery; CSI_10 to CSI_13 on the factor of Prototyping and CSI_14 to CSI_19 on the factor of Evaluation. Residual errors (e) indicated as follows: e1 to e5 corresponding to the items on Visualization; e6 to e8 to the items on Discovery; e9 to e12 to the items on Prototyping; and e13 to e18 to the items on Evaluation

To further examine whether the four DT components would converge on one single, unitary construct, a second-order CFA model with the four factors loaded on the latent factor of ‘DT competence’ was performed. The standardized coefficients for the indicators and the latent variables in the model are shown in Fig. 4. Results revealed that the goodness-of-fit indices for the model were: χ^2^/df = 4.082, NNFI = 0.935, CFI = 0.949, TLI = 0.940, SRMR = 0.040, and RMSEA = 0.080. On the basis of the same evaluation criteria described above for the first-order CFA, the data also showed a good model fit. Results of the second-order confirmatory factor analysis on the 18-item Taiwanese version of Creative Synthesis Inventory, with standardized factor loadings of evaluation, discovery, prototyping, and visualization on the construct of design thinking competence.

#### Comparing first- and second-order CFA models

Given that the the first-order CFA model was nested within the second-order CFA model (Brown, 2006) [37], the chi-square difference test (χ^2^_diff_. = χ^2^_second−order_ - χ^2^_first−order;_ df^2^_diff_. = df^2^_second − order_ - df^2^_first − order_) was used to evaluate which model fit the data better [31]. Results revealed that the value for χ^2^_diff_. was 0.13 and the value of df^2^_diff_. was 2. The chi-square table was then checked to see whether the p-value for χ^2^_diff_. was at the significant level of 0.05. The calculated p-value of 0.94 indicated that there was no significant difference between the models. Thus, the second-order CFA model was acceptable.

### Convergent and discriminant validity

To test the convergent validity of CSI-Taiwan, composite reliability (CR) and the average variance extracted (AVE) were computed. Results of the computed CRs and AVEs for the four (latent) components of the CFA model are displayed in Table 4. Given that the values of CRs, ranging from 0.910 t0 0.933, were greater than 0.70 and that the values of AVEs, ranging from 0.673 to 0.771, were greater than 0.50, the convergent validity of the 18-iem CSI-Taiwan was demonstrated. To test the discriminant validity of CSI-Taiwan, square root of AVEs and inter-construct correlation coefficient were computed. Table 4 showed that the square root of the AVEs for the four components, ranging from 0.821 to 0.933, were higher than its correlation with other component, which provided evidence for the discriminant validity.

**Table 4:** Results of convergent and discriminant validity tests for the 18-item Taiwanese version of Creative Synthesis Inventory

	CR (ω)	AVE	Correlation coefficients			
**Component**			Visualization	Discovery	Proptyping	Evaluation
Visualization	0.911	0.673	**0.821**			
Discovery	0.910	0.771	0.726^**^	**0.878**		
Proptyping	0.911	0.720	0.729^**^	0.607^**^	**0.911**	
Evaluation	0.933	0.700	0.766^**^	0.735^**^	0.789^**^	**0.933**

Note: CR = Composite reliability indexed by omega (ω); AVE = Average variance extracted; square root of AVEs are shown on diagonal (highlighted with bald font)

### Reliability: internal consistency

The reliability indexed by Cronbach’s alpha of the 18-item CSI-Taiwan was 0.876, 0.907, 0.917, and 0.930 for Visualization, Discovery, Prototyping, and Evaluation, respectively. Its internal consistency was 0.961 for the total scale of DT Competence. Together, these values greater than the recommended level of 0.80 indicated that the reliability of the CSI-Taiwan was good.

## Discussion

The current study employed a vigorous process of item examination, involving forwards- and backwards-translation as recommended by Brislin [26] as well as expert reviews for the content and face validity of scale items. Psychometric characteristics of the Taiwanese version of CSI further underwent rigorous quantitative evaluations. Based on the results of reliability examination as well validity tests, it was concluded that the CSI-Taiwan had (a) good reliability indexed by internal consistency; (b) satisfactory construct validity based on the values of factor loadings, CRs and AVEs; (c) confirmation of a theorized 4-factor structure as evidenced by the findings of a first-order CFA; and (d) demonstration of an overarching, unitary construct of “Design Thinking Competence” as supported by the acceptable model fit of a second-order CFA.

Several studies have shown the reliability and validity of measurements when assessing DT competence in the students of higher education [31, 38]. However, it should be noted that the components in the process of DT has not been extensively studied. Only a few studies on the creative synthesis process within DT environments have been conducted worldwide [9]. To the best of our knowledge, no study has performed translation and cultural adaptation of an instrument evaluating DT competence. CSI-Taiwan was the first cross-culturally tested and validated instrument designed for measuring nursing students’ DT competence. In addition to employing a relatively large sample ( *n* = 520), the major advancement of the current study on the cross-cultural adaptation of CSI to the healthcare field was in a rigorous quantitative evaluation of the scale’s psychometric properties. Specifically, advanced statistical techniques of first- and second-order CFAs were used to test construct validity. Multiple indices were applied to assess the convergent and discriminant validity of CSI-Taiwan.

Both the reliability and validity of CSI-Taiwan were supported with strong evidence. First, the first-order CFA confirmed a four-factor structure that was consistent with Badding’s theorization [9], which describes the creative synthesis process within a DT environment consisted of the components of visualization, discovery, prototyping, and evaluation. The correlations between the four components of creative synthesis skills in the CFA model also substantiated the conceptualization that DT involves a non-linear, dynamic, and iterative process [1, 12].

One, however, should note that an item (i.e., CSI_9: Test and define to gain feedback and learn rapidly) was removed from the Discovery component due to its low factor loading. One explanation for this finding could be due to the instability of Discovery component in the creative synthesis process. As noted by Badding [9], the Discovery component was a newly emerged construct during the empirical construction of CSI. The existence of Discovery component in the DT environments was questioned. Together with the finding from the current study, one conclusion could be reached was that the Discovery skills perhaps are prevalent in the DT process, but the specific skills involved need to be further examined. An alternative explanation, which also provide further support for the first explanation, was that while the other three items on Discovery component specifically highlighted the competence of collaboration with others, the removed item of CSI_9 captured a dual construct of collaboration and fast learning. Specifically, while the first part of this item tapped into the construct of collaboration, its second part focused on rapid learning. While collaboration with others might enhance the breadth and depth of learning, the nature of collaborative work, in fact, often impedes the speed of learning. The contradictory duality in CSI_9 might nullify its construct validity.

Still, taking a cross-cultural perspective, there is one more possible explanation for why CSI_9’s emphasis on feedback-seeking appeared to be invalid with Taiwanese students. It is long been argued that significant cross-cultural differences exist in individuals’ beliefs, norms, values, and self-construal’s between those in individualistic-independent cultures (e.g., United States) and those in collectivistic-interdependent cultures (e.g., Taiwan) [39]. Whereas self-enhancement tendencies are more prominent among people in individualistic-independent cultures, self-improvement tendencies are far more common among individuals in collectivistic-interdependent cultures [40]. Stone-Romero and Stone [40] further theorized that individuals’ response to feedback differs according to their cultural background. Those in individualistic-independent cultures view feedback, particularly, negative performance feedback, as a threat and react with an array of self-defensive strategies, such as deny, ignore, and/or avoidance, to maintain and enhance self-esteem. By contrast, those in collectivistic-interdependent cultures view feedback as a valuable resource for self-enhancement. As such, Taiwanese students who may differ from their American counterparts participating in the original CSI study, failed to view feedback-seeking as a unique and valuable discovery skill learned from the capstone course that can promote their DT competence.

Finally, the second-order CFA further confirmed that the four components of creative synthesis skills converged on a single, unitary construct of DT competence. Such finding may suggest that DT competence covers the totality of evaluation, discovery, prototyping, and visualization, which is consistent with a process view of DT.

## Limitations

This study has several limitations. First, a methodological concern of this research is that sampling students from one university may limit the external validity of our findings. Future studies recruiting students from multiple universities is recommended. Second, criterion-validity was not assessed in this study because a gold-standard criterion instrument is not available. Regarding discriminant validity, no additional instruments were included in this study to facilitate the evaluation. Hence, future research should include other measures that are known to be conceptually similar or different from DT competence. For example, an objectively evaluated DT competence, such as students’ grade for the capstone course, could be used as a measure for assessing convergent validity. Third, the participants were selected from those who enrolled in the capstone course offered to students nearing the end of their 2- or 4-year program. Whereas the course was offered to the second-year students of the 2-year program, it was offered to the third-year students of the 4-year program. The characteristics of the target participants may have varied with their programs and school years. Future studies need to improve the homogeneity of the participants, targeting students in the same school year and program. It may also worthy noting that the data collection of this study spanning from before to during the COVID-19 time period. Non-face-to face classroom situations during the COVID-19 time period may have affected the students’ self-perceived DT competence. Finally, the CSI-Taiwan was only validated with nursing students, which should be further tested for its use with healthcare professionals for a wider application.

## Conclusions

The progress of DT research has been challenged by a lack of agreement on definitions and instruments. Guided by the related theories and empirical studies, this study followed a best practice of mixed methodological design combining qualitative and quantitative strategies. This cross-culture adaptation research validated an instrument assessing DT competence in nursing students. Rigorous psychometric analysis has demonstrated CSI-Taiwan was a valid and reliable scale to be used with Taiwanese college students in nursing. Additionally, the conceptual framework that guided the study can be used to enhance healthcare professionals’ understanding of DT and its core components.

### Electronic supplementary material

Below is the link to the electronic supplementary material.

Supplementary Material 1

## Data Availability

The datasets used and/or analyzed during the current study are available from the corresponding author on reasonable request.

## Abbreviations

DT: Design thinking
CSI: Creative Synthesis Inventory
DTTQ-TW: Design Thinking Traits Questionnaire
